# Bridging the Gap Between Family Medicine and Adolescents: Creating Opportunities Through Sex Education

**DOI:** 10.7759/cureus.31472

**Published:** 2022-11-14

**Authors:** Miguel Abreu, Cármen Silva, Mégane Vieira, Romeu Soares, Luísa Pinheiro

**Affiliations:** 1 Family Medicine, Unidade de Saúde Familiar Martim - Agrupamentos de Centros de Saúde (ACeS) Cávado III, Barcelos, PRT; 2 Family Medicine, Unidade de Saúde Familiar Arquis Nova - Unidade Local de Saúde do Alto Minho, Viana do Castelo, PRT; 3 Family Medicine, Unidade de Saúde Familiar Vale do Sorraia - Agrupamentos de Centros de Saúde (ACeS) Leziria, Santarém, PRT; 4 Family Medicine, Unidade de Saúde Familiar Viatodos - Agrupamentos de Centros de Saúde (ACeS) Cávado III, Barcelos, PRT

**Keywords:** family planning, contraception literacy, contraception, high school students, sexual literacy, sex education, adolescents

## Abstract

Introduction: Despite school sex education programs defined by law and a national healthcare service with dedicated family planning appointments, the number of teenage pregnancies in Portugal remains significant. The implementation of these programs has been found lacking and a disconnection between adolescents and primary healthcare has been identified. Adolescents have low literacy in contraception, with males being less involved and informed. With this project, the authors sought to propose an alternative approach to school sex education, aiming to improve the global knowledge and adequate use of contraception methods by teenagers.

Material and Methods: Single-arm prospective interventional study was done to assess the impact of a new model of sexual education learning on adolescents’ knowledge and use of contraception methods. A 30-minute lecture was developed by Family Medicine residents focusing on the main aspects of contraception and sexual education, using informal language and slideshow presentations. A form using “Yes/No”, “True or False”, multiple answer, and Likert scale questions was built to assess attitudes and levels of literacy. Two time points were defined to assess the impact of the intervention, applying the form before and one month after the lecture. Four high schools were invited to enter the study, with 190 students enrolled after personal and guardian authorization. IBM SPSS Statistics for Windows, Version 27.0 (Released 2020; IBM Corp., Armonk, New York, United States) was used to perform statistical analysis.

Results: A total of 190 participants aged between 14 and 19 years answered both forms. A high 73.7% reported never having had a family planning appointment, with “Not knowing about the possibility” and “Not feeling comfortable with” being the main reasons; 26.3% were sexually active, from which 44% admitted having had intercourse without any kind of protection. Regarding sources of information about contraception, “School,” “Internet,” and “Friends” were the most prevalent, with only 43% considering the healthcare providers a source. In the contraceptive literacy evaluation, the participants overestimated their knowledge pre-intervention; however, this gap was attenuated in the second evaluation. Moreover, there was a statistically significant improvement in literacy between assessments for all genders. After the intervention, 40.6% reported discussing contraception outside the classroom.

Discussion: The low attendance of the family planning appointments and the low number of participants that considered healthcare services as a source of information in contraception conveys the idea that primary healthcare does not play the desired role in disseminating reliable information on contraception among teenagers. This is concerning considering the overestimated knowledge, low literacy, and risky behaviors identified. The intervention managed to improve the teenagers’ literacy while reinforcing the importance of family planning appointments and inciting dialogue among the participants.

Conclusion: This project presents an alternative to current school sex education models and focuses on streamlining communication by resorting to younger communicators, scientific but informal messaging, and short sessions. While extended research comparing against the set models needs to be done, it poses an opportunity to bring adolescents closer to primary healthcare while gathering data to improve clinical practice.

## Introduction

In Portugal, the average age of sexual debut ranges from 14 to 15.6 years [1]. The Portuguese National Health Service provides for sexual health information and support at the primary care level, both in regular routine consultations, included in the National Child and Youth Health Plan, and in family planning appointments [2]. The latter are intended for all people (regardless of gender) over 15 years of age, are available free of charge, and are carried out in partnership between the nursing and medical staff. In addition to information leaflets, most contraception is provided at no cost (including emergency contraception) [2]. Furthermore, sex education classes are mandatorily included in all school programs (from elementary to high schools), since 2009. This law applies to all educational establishments, whether public or private [3]. However, in a 2019 report, upon monitoring and evaluation of the implementation of the law, it was found that most schools do not adequately comply with the sex education program [4].

Despite the above, in 2020 Portugal ranked 11th in absolute numbers of teenage pregnancy live births among European countries, with teenage pregnancies amounting to 2.1% of all live births in the country [5,6]. In 2014, only one-third of Portuguese teenagers had ever visited a healthcare facility seeking contraception counseling and less than fifty percent had ever attended classes on reproductive health [1,7,8].

Regarding contraception literacy in adolescents, international studies show that while being generally low, it is higher among female teenagers, and males are often less involved and informed about contraceptive methods [9-12].

Bearing in mind the status of teenage pregnancy in Portugal, the consequences of low sexual health literacy, and the discrepancies among genders described in the literature, the authors sought to identify and assess local teenagers’ knowledge, attitudes, doubts, and needs regarding contraception while proposing an alternative approach to sexual health education.

## Materials and methods

Approval and eligibility 

The project obtained approval from the Institutional Review Board at Unidade Local de Saúde do Alto Minho, Portugal, (approval number 35/2022) and authorization from the Ministry of Education (request number 0823400001) and school boards. The support of the local school health teams was attained and the project developed with their cooperation. Four high school institutions were invited and all classes that had no intervention on the subject during the year the project took place were eligible. Written authorization from both the participant and legal guardian was required to enroll.

Intervention and questionnaire 

A single-arm prospective interventional study was created, with the intervention designed to be conducted by Family Medicine residents. The authors hypothesize that the smaller age gap and being external to school would reduce the communication barrier. Slideshow presentations on contraception were held focusing on scientific but informal communication; the themes approached were the physiology of fertility and menstrual cycle, contraceptive choice and mechanism of action, and the works of the Portuguese National Health Service. The presentations were intended to be short - 30 minutes.

Two contacts were made with the classes: in the first contact, an anonymous, original questionnaire was applied to characterize the sample demographics and healthcare usage and gauge the general knowledge about contraception, followed by a presentation on the theme. On a second visit, circa one month later, the participants were asked to answer the same questionnaire and a new presentation was held, focusing on participants’ doubts and most common mistakes.

The questionnaire, created for the purpose of the project, consisted of three parts: the first one with “Yes or no”, multiple answer, and Likert scale questions to characterize demographics and behaviors of the participants; the second part with 25 true or false questions of varying difficulty evaluating healthcare use and knowledge of contraceptive methods regarding use, efficacy, and differences; the third part with an open answer question for participants to write their doubts and suggestions regarding clinical practice and contraceptive use among youth.

Statistical analysis 

Statistical analysis was performed with recourse to IBM SPSS Statistics for Windows, Version 27.0 (Released 2020; IBM Corp., Armonk, New York, United States). The sample normality was assessed through histograms and normal probability plots. To validate the distribution assessment, Kolmogorov-Smirnov tests were used. The Wilcoxon signed-rank test and Mann-Whitney U test were used to compare, respectively, a single sample with an aim score and the difference between the two samples. The threshold for statistical significance was considered p<0.05. 

## Results

Sample demographics, knowledge, and attitude toward contraception**

Four high school institutions were invited, from which three accepted the invitation. From these, all the eligible classes from 10th, 11th, and 12th grades were included, which comprised 198 students. Eight students had no guardian authorization to participate. The remaining 190 were enrolled as participants whose age, grade, and gender identity (the participants identified as Male - M, Female - F, and Non-Binary - NB) are described in Table 1. 

**Table 1:** Age, gender, and grade sample distribution

		Male	Female	Non-Binary	Total
Age, mean (range) years		16.7 (15-19)	16.7 (15-19)	14	16.7 (14-19)
Grade, n (%)	10	44 (23.2)	27 (14.2)	1 (0.5)	72 (37.9)
	11	10 (5.3)	6 (3.2)	0 (0)	16 (8.4)
	12	50 (26.3)	52 (27.4)	0 (0)	102 (53.7)
Total, n (%)		104 (54.7)	85 (44.7)	1 (0.5)	190 (100)

When asked if they ever had a family planning appointment, 26.3% answered “Yes” (M 18.3%, F 36.5% and NB 100%). The main reasons indicated for never having had an appointment were: “Not knowing about it,” “Not feeling comfortable with,” and, for the male sample, “Not considering it important.”

Regarding sexual relationships, 26.3% (M 24%, F 29.4%, and NB 0%) were sexually active. From these, all admitted to using contraception methods regularly; however, 44% (M 40%, F 48%, and NB 0%) had already had intercourse without any kind of protection. 

**Figure 1:**
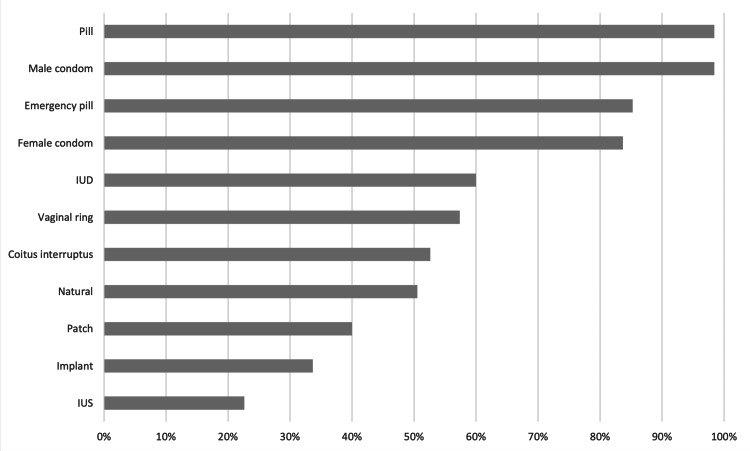
“Which methods do you have any knowledge about” Answers displayed in percentage of total participants (n=190). Multiple answers could be chosen. IUD – Intrauterine Device; IUS – Intrauterine System

The main sources of information regarding contraception were “School” (85%), “Internet” (77%), and “Friends” (69%), with only 43% considering the healthcare providers a source of information. The answers to the question “Which methods do you have any knowledge about” are presented in Figure 1, the most well-known being condoms, oral contraceptives ("pill"), and emergency contraception.

**Figure 2:**
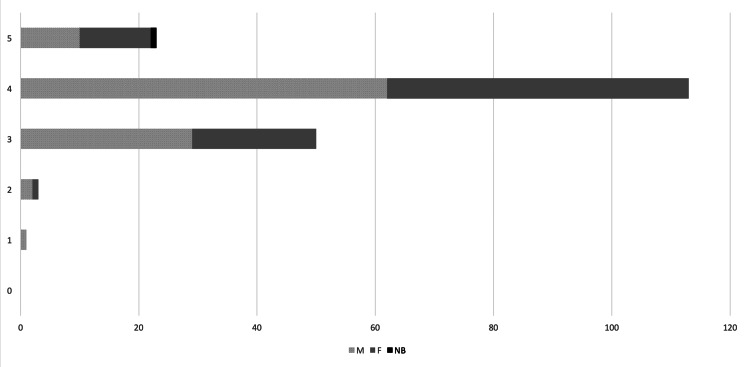
“How do you rank your knowledge on contraceptives” Absolute number of answers per gender – M - Male, F - Female, N - Non-Binary. Possible answers ranged from 0 - “Insufficient” to 5 - “Excellent”

**Figure 3:**
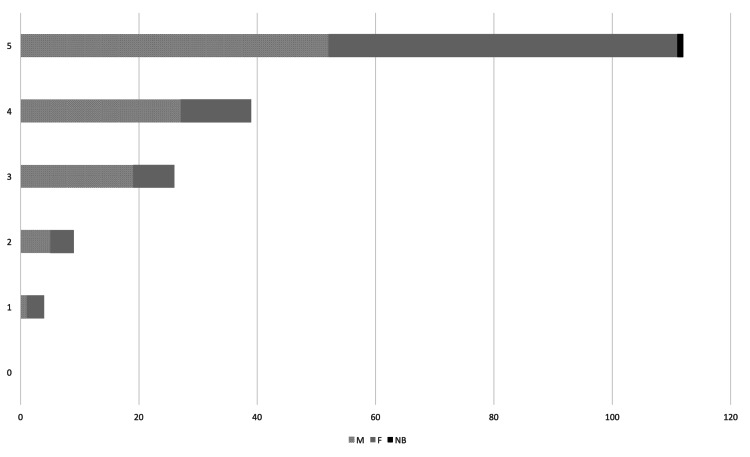
“I would like to know more about contraception” Absolute number of answers per gender – M - Male, F - Female, NB - Non-Binary. Possible answers ranged from 0 - “Completely disagree” to 5 - “Completely agree”

Most participants classified their knowledge of contraceptives close to "excellent" (Figure 2), with, on a scale from 0 to 5, an average of 3.81 (M 3.75, F 3.88, NB 4). However, the majority of the participants, also stated they wished to know more about the theme (Figure 3).

Finally, as a way of gauging the male approach to contraception, the male sample was asked: “Contraception is mainly focused on women and, therefore, not a thing I worry about”, to which, only 5.7% agreed with.

Questionnaire results 

To evaluate attitudes and knowledge on contraception, a 25-question evaluation was applied. The questions and results from each session are presented in Table 2. 

**Table 2:** Questionnaire results presented in number and percentage of correct answers per gender in both assessments The correct answer is presented after the question. STI – Sexually Transmitted Infection

	Pre-Intervention				Post-Intervention			
	Male, n (%)	Female, n (%)	Non-Binary, n (%)	Total, n (%)	Male, n (%)	Female, n (%)	Non-Binary, n (%)	Total, n (%)
Attitudes	198 (95.2)	168 (98.8)	2 (100)	368 (96.9)	203 (97.6)	170 (100)	2 (100)	375 (98.7)
Contraception is a woman’s responsibility - false	97 (93.3)	84 (98.8)	1 (100)	182 (95.8)	102 (98.1)	85 (100)	1 (100)	188 (98.9)
Only women should be informed about contraception - false	101 (97.1)	84 (98.8)	1 (100)	186 (97.9)	101 (97.1)	85 (100)	1 (100)	187 (98.4)
Healthcare and Contraception Access	160 (76.9)	130 (76.5)	2 (100)	292 (76.9)	193 (92.8)	162 (95.3)	2 (100)	357 (93.9)
The National Health Service provides free contraception - true	70 (67.3)	61 (71.8)	1 (100)	132 (69.5)	97 (93.3)	81 (95.3)	1 (100)	179 (94.2)
Man can have Family Planning appointments - true	90 (86.5)	69 (81.2)	1 (100)	160 (84.2)	96 (92.3)	81 (95.3)	1 (100)	178 (93.7)
General Contraception Knowledge	100 (48.1)	112 (65.9)	1 (50.0)	213 (56.1)	146 (70.2)	150 (88.2)	2 (100)	298 (78.4)
The use of contraception causes infertility - false	75 (72.1)	67 (78.8)	1 (100)	143 (75.3)	86 (82.7)	81 (95.3)	1 (100)	168 (88.4)
Hormonal contraceptives (as the pill) are safe for teenagers - true	25 (24.0)	45 (52.9)	0 (0)	70 (36.8)	60 (57.7)	69 (81.2)	1 (100)	130 (68.4)
Condoms	372 (89.4)	320 (94.1)	4 (100)	696 (91.6)	403 (96.9)	331 (97.4)	4 (100)	738 (97.1)
The male and female condoms protect against STIs - true	100 (96.2)	84 (98.8)	1 (100)	185 (97.4)	97 (93.3)	82 (96.5)	1 (100)	180 (94.7)
Condoms have an expiration date - true	88 (84.6)	81 (95.3)	1 (100)	170 (89.5)	103 (99.0)	85 (100)	1 (100)	189 (99.5)
To prevent pregnancy, condoms only need to be used right before the ejaculation - false	82 (78.8)	71 (83.5)	1 (100)	154 (81.1)	100 (96.2)	79 (92.9)	1 (100)	180 (94.7)
Condoms can be used more than once - false	102 (98.1)	84 (98.8)	1 (100)	187 (98.4)	103 (99.0)	85 (100)	1 (100)	189 (99.5)
Pill	101 (48.6)	114 (67.1)	1 (50.0)	216 (56.9)	163 (78.4)	161 (94.7)	2 (100)	326 (85.8)
The pill protects against STIs - false	81 (77.9)	82 (96.5)	1 (100)	164 (86.3)	90 (86.5)	84 (98.8)	1 (100)	175 (92.1)
There is no drop-off in pill efficacy if it is taken in the 12 hours after the usual time - true	20 (19.2)	32 (37.6)	0 (0)	52 (27.4)	73 (70.2)	77 (90.6)	1 (100)	151 (79.5)
Vaginal Ring	26 (12.5)	36 (21.2)	0 (0)	62 (16.3)	66 (31.7)	70 (41.2)	1 (50.0)	137 (36.1)
The vaginal ring should be placed only by healthcare professionals - false	17 (16.3)	26 (30.6)	0 (0)	43 (22.6)	45 (43.3)	52 (61.2)	1 (100)	98 (51.6)
The vaginal ring works by blocking the passage of sperm - false	9 (8.7)	10 (11.8)	0 (0)	19 (10.0)	21 (20.2)	18 (21.2)	0 (0)	39 (20.5)
Transdermal Patch	14 (13.5)	29 (34.1)	0 (0)	43 (22.6)	47 (45.2)	60 (70.6)	0 (0)	107 (56.3)
The transdermal contraceptive is a patch that should only be changed when it falls off - false	14 (13.5)	29 (34.1)	0 (0)	43 (22.6)	47 (45.2)	60 (70.6)	0 (0)	107 (56.3)
Implant	22 (21.2)	43 (50.6)	0 (0)	65 (34.2)	65 (62.5)	75 (88.2)	1 (100)	141 (74.2)
The subcutaneous implant is a long-term contraceptive method that can be placed in general practice clinics - true	22 (21.2)	43 (50.6)	0 (0)	65 (34.2)	65 (62.5)	75 (88.2)	1 (100)	141 (74.2)
Intrauterine contraception	51 (24.6)	98 (57.7)	0 (0)	149 (39.2)	128 (61.5)	151 (88.8)	2 (100)	281 (73.9)
The intrauterine system/device should only be placed by a trained healthcare provider - true	40 (38.5)	60 (70.6)	0 (0)	100 (52.6)	68 (65.4)	80 (94.1)	1 (100)	149 (78.4)
The intrauterine system/device should only be used by women that have already been pregnant - false	11 (10.6)	38 (44.7)	0 (0)	49 (25.8)	60 (57.7)	71 (83.5)	1 (100)	132 (69.5)
Natural Methods	102 (49.1)	107 (63.0)	1 (50.0)	210 (55.3)	141 (67.8)	151 (88.8)	2 (100)	294 (77.4)
A pregnancy cannot occur if the intercourse occurs during menstruation - false	54 (51.9)	59 (69.4)	0 (0)	113 (59.5)	65 (62.5)	79 (92.9)	1 (100)	145 (76.3)
The withdrawal method is effective as long as the ejaculation doesn’t occur inside the woman’s body - false	48 (46.2)	48 (56.5)	1 (100)	97 (51.1)	76 (73.1)	72 (84.7)	1 (100)	149 (78.4)
Permanent Contraception	43 (20.7)	86 (50.6)	0 (0)	129 (34.0)	70 (33.7)	112 (65.9)	2 (100)	184 (48.4)
Vasectomy denies the ejaculation/orgasm - false	20 (19.2)	42 (49.4)	0 (0)	62 (32.6)	38 (36.5)	62 (72.9)	1 (100)	101 (53.2)
After tubal ligation women enter menopause - false	23 (22.1)	44 (51.8)	0 (0)	67 (35.3)	32 (30.8)	50 (58.8)	1 (100)	83 (43.7)
Emergency Contraception	74 (23.7)	103 (40.4)	0 (0)	177 (31.0)	151 (48.4)	187 (73.3)	3 (100)	341 (59.8)
Emergency Contraception acts in a similar way to an abortion - false	19 (18.3)	28 (32.9)	0 (0)	47 (24.7)	44 (42.3)	65 (76.5)	1 (100)	110 (57.9)
The “plan-b” pill can be regularly used as an alternative to regular contraception - false	41 (39.4)	67 (78.8)	0 (0)	108 (56.8)	52 (50.0)	60 (70.6)	1 (100)	113 (59.5)
There is a limit of “plan-b” pills than can be taken in a lifetime - false	14 (13.5)	8 (9.4)	0 (0)	22 (11.6)	55 (52.9)	62 (72.9)	1 (100)	118 (62.1)
Total	1263 (48.6)	1346 (63.3)	11 (44.0)	2620 (55.2)	1776 (68.3)	1780 (83.8)	23 (92.0)	3579 (75.3)

In the pre-intervention assessment, our sample evidenced a high literacy regarding the usage of condoms (91.6%) and a positive attitude across all genders towards contraception use and shared responsibility. There was low knowledge of all the other contraceptive methods, with only the pill and natural methods achieving more than 50% of correct answers. Concerning safety, 75.3% knew that the use of contraception is not associated with infertility; however, only 36.8% believed that hormonal contraceptives are safe for teenagers. 

Overall, the sample scored 55.2% of correct answers with the female sample scoring higher (63.3%) than the male (48.6%) and non-binary (44%). The difference between the male and female samples was statistically significant (p<0.01). The authors deemed the non-binary sample too small (n=1) to be representative of the population, therefore opting to not include it in the comparison statistics. 

In the open-answer section of the form and the question-and-answer (Q&A) session at the end of the presentation, regarding doubts and suggestions, the participants posed a wide array of questions ranging from sexually transmitted infections (STIs) prevention to contraception prices and access. The main themes and frequency of the questions are presented in Table 3. 

**Table 3:** Questions placed in the open-answer section and Q&A grouped by theme STI – Sexually Transmitted Infection, Q&A – Questions and Answers

Questions	n (%)
STIs transmission and prevention in various types of intercourse	15 (22.7)
Access to free contraceptives in the National Healthcare System	14 (21.2)
Family planning appointment	12 (18.2)
Emergency contraception	9 (13.6)
Information about contraceptive methods less commonly spoken about	8 (12.1)
Contraception and prevention of STIs in non-heterosexual relationships	5 (7.6)
Natural methods	3 (4.5)
Total	66 (100)

Effects of the intervention on knowledge and attitudes 

After the intervention, the overall score was higher (75.3%), with the female score remaining higher (83.8%) than the male (68.3%). The non-binary participant improved to a score of 92%. The improvement (total and per gender) between questionnaires and the difference between the male and female scores in the second evaluation were statistically significant (p<0.01). The comparisons of the samples and moments of evaluation were analyzed using Mann-Whitney U tests.

Based on the participants' self-reported knowledge on a scale from zero to five, we were able to predict the participants' number of correct answers and compare them to the actual results. In the first evaluation, the male sample classified its knowledge on average as 3.75, for an expected score of 18.75 correct answers, which contrasted with the actual score of 12.1 (p<0.01). The females graded their knowledge as 3.88, for an expected score of 19.5, scoring 15.8 (p<0.01). The non-binary sample graded its knowledge as 4, for an expected score of 20, scoring 11.

In the second evaluation, the male, female, and non-binary samples classified their knowledge, respectively, as 3.95, 4.18, and 5. The male expected score was 19.75 versus the actual 17.1 (p<0.01), the female expected score was 20.9 with an actual score of 20.9 (p=0.08), and the non-binary expected score was 25, scoring 23 in this evaluation. As samples did not follow a normal distribution, the value was obtained through single-sample Wilcoxon signed-rank tests.

When asked if contraception was a theme they searched for or discussed among peers or family after the first session, 40.6% of the participants answered “Yes” (M 28%, F 55%, and NB 100%).

Using the open-answer question, some participants (n=12) evidenced the positive outcome of the intervention by commending its relevance and ease of communication during the sessions.

## Discussion

Our results showed that primary healthcare and sex education programs were not playing the desired role in reliably disseminating information on contraception among teenagers. This might be attributed to poorly adapted content, communication methodology, or choice of lecturers, as a recent literature review showed that these were some of the main reasons why adolescents may be discontent with sex education [13].

Only 26.3% of respondents had attended a consultation exclusively dedicated to sexual health education, which includes contraception. This value, while in line with the results described by previous studies [1,7,8], may be overestimated since participants may confuse the family planning consultations with the routine appointments included in the National Child and Youth Health Plan. Moreover, the low number of participants that considered healthcare a source of information should be worrying, as it is known that counseling on contraception provided by health professionals increases its use [1,14].

Assessing their knowledge and attitudes, our male sample contradicted the trend described in the literature as they showed a more positive attitude and a greater sense of responsibility regarding the subject. On the other hand, as expected, the female sample evidenced higher levels of literacy [9-12].

All genders overestimated their level of knowledge on the topic when compared to the actual result obtained in the questionnaire response. According to previous research, a wrongful estimation of one’s knowledge may lead to a wrongful estimation of risks and thus, pose a predisposition to risky behavior - in our sample, 44% of sexually active adolescents admitted to having had sex without any contraceptive method [15]. After the intervention, there was a mitigation of the overestimation, being even nullified in the female sample.

Only one session per class, lasting 30 minutes, was needed to obtain a statistically significant improvement in the sample’s contraception literacy, even if the discrepancy between male and female samples remained. However, the post-intervention male sample achieved better results than the pre-intervention female sample. A bias possibility should be considered, either because the participants may have copied answers among peers and/or because the questions were the same pre- and post-intervention.

Aware of the importance of the theme for adolescents and the difficulties they face in approaching these issues (“not feeling comfortable” was one of the main reasons mentioned for the non-frequency of family planning consultations), the results showed that the participants were interested in knowing more about contraception, which possibly contributed to the positive results. Their curiosity was evidenced in the pre-intervention assessment (the majority answered they wished to know more about contraception), during the intervention as they sought to clarify doubts during the sessions and in the questionnaire (66 oral or written questions were asked), and post-intervention as a significant percentage (40.6%) discussed and/or sought information about the theme after the presentation.

Bringing medical residents to the adolescents' comfort zone, resorting to group presentations, and using scientifically focused presentations leading to easier communication are also possible reasons for the good results obtained [13]. At the beginning of the current school year, students from one of the participant schools asked the representatives to invite the authors to reprise and expand the presentations on sexual health. The authors consider that the project may have helped reduce the barrier between the sample and healthcare providers.

Nonetheless, the present results should be interpreted with the study limitations in mind, viz., the use of an original questionnaire may skew the results as it is possible that the items assessed may not be representative of the real knowledge about contraception; this is a common obstacle in studies regarding contraceptive literacy [16,17]. The study was developed in schools close to the authors’ practice location (two in the Northern region of Portugal and one in the Center) and results may not represent the knowledge and attitudes of adolescents throughout the whole country; however, sexual education programs and family planning appointments are defined by law, so, barring local or regional constraints, all adolescents should have been exposed to similar information.

## Conclusions

The fact that adolescents admit to not knowing about the existence of family planning consultations or feeling ashamed to address their concerns about sex to health professionals highlights a clear need to work towards bringing primary healthcare closer to its users.

This project lays the foundation for an alternative to sex education projects currently in place, benefiting from promoting proximity between healthcare providers and adolescents while improving literacy and gathering data to adapt the clinical practice in this age range. There remains, however, the necessity to scientifically compare the different approaches, while working to expand to other relevant sexual health topics.
